# The Benefits of Unnatural Amino Acid Incorporation as Protein Labels for Single Molecule Localization Microscopy

**DOI:** 10.3389/fchem.2021.641355

**Published:** 2021-03-25

**Authors:** Pooja Laxman, Shirin Ansari, Katharina Gaus, Jesse Goyette

**Affiliations:** European Molecular Biology Laboratory (EMBL) Australia Node in Single Molecule Sciences, School of Medical Sciences, University of New South Wales, Sydney, NSW, Australia

**Keywords:** unnatural (non-canonical) amino acids, single molecule localization microscopy, fluorescent protein, unnatural amino acid incorporation, stochastic optical reconstruction microscopy, photo-activated localization microscopy, self-labeling protein tag

## Abstract

Single Molecule Localization Microscopy (SMLM) is an imaging method that allows for the visualization of structures smaller than the diffraction limit of light (~200 nm). This is achieved through techniques such as stochastic optical reconstruction microscopy (STORM) and photoactivated localization microscopy (PALM). A large part of obtaining ideal imaging of single molecules is the choice of the right fluorescent label. An upcoming field of protein labeling is incorporating unnatural amino acids (UAAs) with an attached fluorescent dye for precise localization and visualization of individual molecules. For this technique, fluorescent probes are conjugated to UAAs and are introduced into the protein of interest (POI) as a label. Here we contrast this labeling method with other commonly used protein-based labeling methods such as fluorescent proteins (FPs) or self-labeling tags such as Halotag, SNAP-tags, and CLIP-tags, and highlight the benefits and shortcomings of the site-specific incorporation of UAAs coupled with fluorescent dyes in SMLM.

## Introduction

Advances in fluorescence microscopy have now allowed researchers to investigate the intricate details behind subcellular protein localization and organization. However, these methods were limited in optical resolution due to the diffraction limit of light microscopy (Adhikari et al., 2019). Single Molecule Localization Microscopy (SMLM) belongs to the subset of super-resolution imaging and has positively impacted the ways in which cell architecture (Jimenez et al., 2020). In conventional microscopy the ability to resolve fluorescent signals arising from molecules in close proximity is limited by the diffraction of photons as they emanate from the point source, pass through the microscope, and are detected by the camera. The point spread function (PSF) of a microscope refers to this “blurring” effect, which limits the ability to distinguish structures on the scale of half the wavelength of the photons that are detected. In a sample with densely packed molecules that are fluorescently tagged, differentiating two molecules that overlap in each other's PSF would render it impossible to resolve through regular light microscopy (Jradi and Lavis, 2019).

Single Molecule Localization Microscopy is able to bypass the resolution limit through the precise localization of individual fluorophores to increase the spatial resolution of single molecules (Xu et al., 2017). This super-resolution is largely achieved through stochastically activating a small subset of fluorophores at any given time. This factor differentiates the technique from regular fluorescence microscopy which do not involve selectively turning the probes “on” and “off” (Hess et al., 2006). The ability to map out detailed images beyond the distances provided by the diffraction limit makes this advantageous over light microscopy techniques to visualize key cellular structures. Through the use of the various SMLM techniques, it is able to detect structures as small as 20 nm in size (Fürstenberg and Heilemann, 2013).

**Figure 1:**
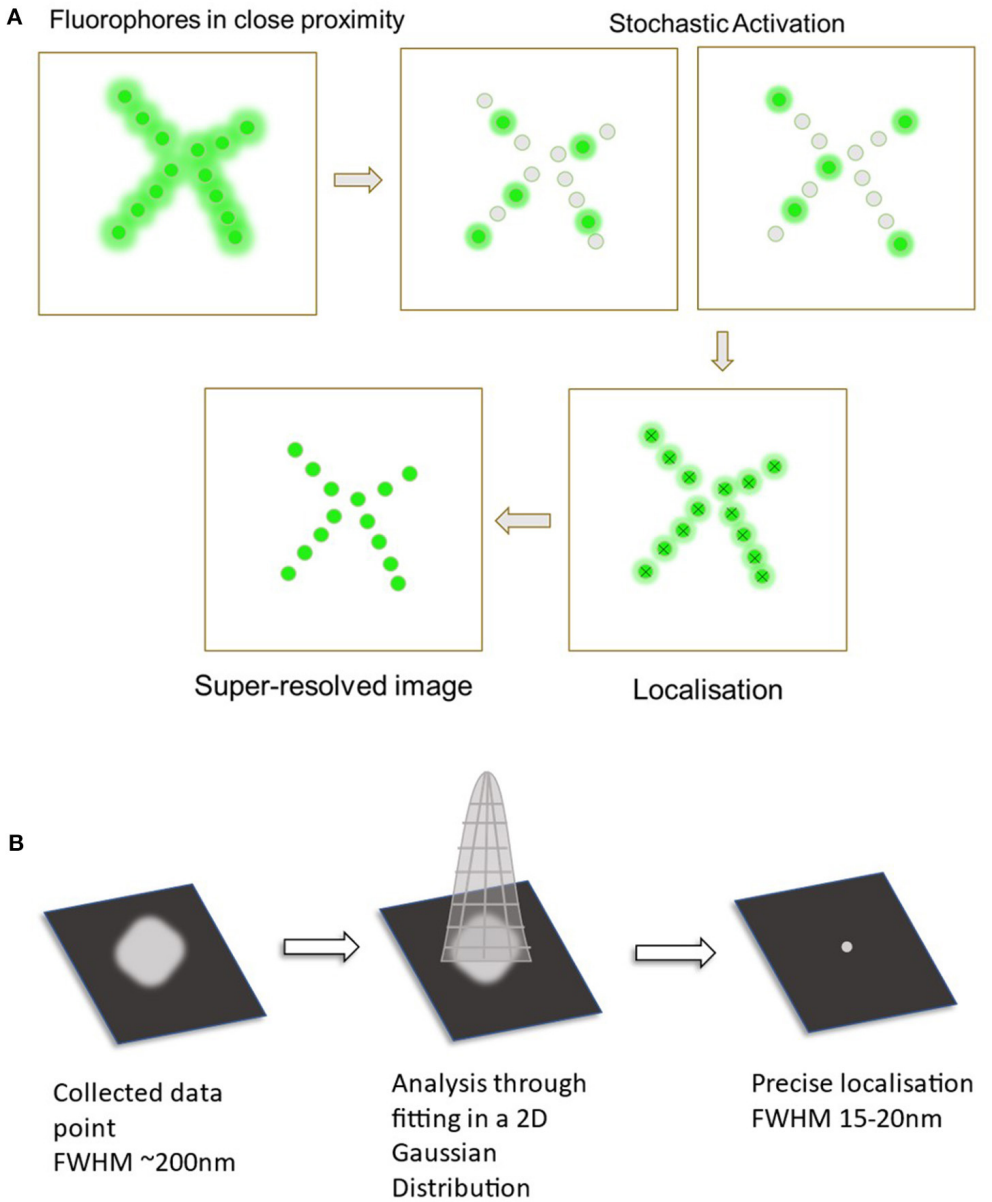
Diagram displaying the process of single molecule localization microscopy (SMLM). **(A)** The primary steps in SMLM starts off with the fluorophores in a densely packed area. These are then stochastically activated to spatially separate the fluorophores. Data from all of the probes are collected and analyzed to localize all the points. This now creates the final super-resolved image. **(B)** Analysis through fitting to a 2D Gaussian distribution. Collected fluorescent signal (Full width at half maximum ~200 nm).

While several techniques are used in SMLM imaging, two primary approaches include Direct Stochastic Optical Reconstruction Microscopy (dSTORM) and Photoactivated Localization Microscopy (PALM) (Almada et al., 2015). These involve the use of reversibly photoswitchable fluorophores that can activate and deactivate periodically (Jimenez et al., 2020). The use of these techniques can allow for the localization of these individual fluorophores with a much higher precision in contrast to regular fluorescence microscopy techniques. Once all the fluorophore points have been collected, single molecule localization can then be achieved through computational analysis (Babcock and Zhuang, 2017). The PSF of each fluorophore is fitted onto a 2D Gaussian curve function that is then used to estimate the exact coordinates of each individual fluorophore (Huang et al., 2008). This together can generate the super-resolution image required to visualize nanoscopic structures and protein-protein interactions (as seen in Figure 1).

In a standard stochastic optical reconstruction microscopy (STORM) experiment, the vital elements required include the use of fluorescent probes to be fused to the target of interest, the separation, and stochastic activation of the individual fluorophores to ensure the proper imaging of single molecules and the localization precision of the fluorophores (Endesfelder and Heilemann, 2015; Xu et al., 2017). The spatial separation of active fluorophores in STORM ensures the activated fluorophores do not overlap their point-spread functions (Adhikari et al., 2019). Photoactivated Localization Microscopy (PALM) similarly shares these features with STORM. What differs the two techniques are the type of fluorescent labels used. Unlike STORM that requires fluorescent probes, PALM makes use of photoactivatable fluorescent proteins (FPs) as the labeling technique (Almada et al., 2015). To control the selective activation of the fluorophores, STORM uses specialized buffers to trap fluorophores in the dark state and low levels of UV light to stochastically photo convert a small subset into the fluorescent state (Betzig et al., 2006). In PALM photoactivatable FPs are stochastically converted from a dark state, or spectral properties of photoswitchable FPs stochastically shifted, by the use of UV light, thereby allowing a subset to be imaged and bleached with high laser power before the next subset is converted (Betzig et al., 2006).

In the context of proteins, a large component of successful SMLM imaging is the choice of fluorescent probe used as a label for the protein of interest (POI). The ideal fluorescent tag candidate for SMLM should aim for photostability during activation and deactivation cycles, be small in size, have high levels of photon emission for better single-molecule detection by the microscope and must not perturb the functionality of the intrinsic interactions of the POI and the other proteins in the environment (Hirabayashi et al., 2011; Toseland, 2013). Various approaches have been developed over the years and have seen great advancement in the creation of photoswitchable fluorophores (Zessin et al., 2012). Types of protein labeling tags include the use of FPs, self-labeling enzymes, and unnatural amino acids (UAAs) (Cranfill et al., 2016; Freidel et al., 2016; Saal et al., 2018). Other forms of labeling such as antibodies are used extensively but have already shown to have come with its own limitations such as the requirement for antibodies with high affinity and high specificity with the target protein. Secondary antibodies are also often used to attach the desired fluorescent dye and can increase technical artifacts in the final image (Li and Vaughan, 2018).

Protein labeling methods that have seen more success and are also used more in recent years include the use of photoswitchable proteins/enzymes that can be genetically fused to the POI (Moerner et al., 2015). This includes the use of naturally FPs (such as GFPs) and self-labeling enzyme tags which aid in attaching the POI with the fluorescent dye for imaging. While several issues come with the use of these labels such as their larger size, the one major limitation that they share is the lack of site-specific tagging (Lee et al., 2019). Therefore, the use of UAAs tagged with a small organic fluorophore can be seen as an attractive alternative approach. The use of gene expansion technology has led to many potential biological applications as the limits to which a gene sequence can be manipulated are theoretically infinite (Ambrogelly et al., 2007). One aspect of this is the use of UAAs as fluorescent markers for SMLM imaging. Through the comparison of the characteristics of all three different types of labeling methods, it is possible to determine that UAA labeling can be a good alternative to the other more commonly used fluorophores (Vreja et al., 2015).

## Types of Labeling Strategies

### Photoswitchable Fluorescent Proteins

In order for particular fluorophores to be utilized in SMLM, a series of reversible photoswitching mechanism is required in order to switch between the fluorescent state and a non-fluorescent state (Li and Vaughan, 2018). Unlike photoactivable FPs which irreversibly shift from an “off” state to an “on” state and are deactivated through photobleaching, photoswitchable FPs provide the ability to shift between two fluorescent states with different excitation and emission profiles over the course of an experiment (Chozinski et al., 2014). Photo-convertible fluorophores could also be a potential alternative as they provide the ability to change their emission spectrum when exposed to UV (Bek et al., 2020). Amongst the choices, reversibly photoswitchable FPs are one of the most common choices for SMLM as the desired fluorophore largely for its efficacy and reliability (Zhou and Lin, 2013). Fluorescent proteins are shown to be useful in discovering the molecular interactions between two species particularly regarding proteins. They can provide further information regarding enzyme activity, protein localization, conformation changes, and many other structural and spatial movements (Toseland, 2013). The photoswitching capability of FPs needed for SMLM can be achieved through the use of specialized buffers or using various levels of UV light (Stiel et al., 2007; Xu et al., 2017). However, despite its widespread usage, it comes with its own disadvantages that can hinder the understanding behind the POI.

Most types of FPs range from 25 to 35 kDa in size and around 4–5 nm in diameter (Crivat and Taraska, 2012). Compared with the localization precision of PALM (<20 nm) this can be seen as bulky and adds to the imprecision of localizing the POI. Furthermore, larger fluorophores could potentially disrupt the natural protein–protein interactions and their general intrinsic activity (Prescher and Bertozzi, 2005). Thus, alternative methods can address this concern by the use of smaller fluorophores to minimize this perturbation. Additionally, due to its size being comparatively larger than other fluorophores, FPs are normally required to be attached to the N or C terminus of the POI to avoid large disruptions of the protein structure or folding behavior (Crivat and Taraska, 2012). Fluorescent proteins additionally do not offer a wide spectral coverage as options for the far-red side of the spectrum become limited (Cranfill et al., 2016).

The structure of a FP consists of a β-sheet barrel that encloses a helix structure containing the central chromophore (Shaner, 2014). While FPs are generally resistant to denaturing, high temperatures and proteolysis, there is variability in the speed of chromophore maturation and in the aggregation of POI-FP fusions (Cranfill et al., 2016). The folding of a FP is essential in maintaining the stability of the chromophore as the β-sheet barrel acts as a protector to the internal chromophore. Perturbation to this structure can cause the loss of the chromophore formation and therefore fluorescence efficiency (Kremers et al., 2011). Finally, FP are typically less bright than organic fluorophores that can be specifically engineered to emit higher levels of fluorescence (Lavis and Raines, 2008).

Fluorescent proteins have been observed to be good candidates as fluorescent tags. However, as it comes with limitations that can alter the results of the finalized super-resolved image, researchers have looked into alternative approaches of labeling.

### Self-Labeling Tags

Self-labeling tags are engineered enzymes that react covalently with their substrates. These substrates can be linked to useful chemical moieties, such as biotin or in the case of SMLM imaging, an organic fluorophore (Liss et al., 2015). Due to the flexibility offered by the available labeled substrates, these tags have been in more common use over the years (Hansen et al., 2016). Examples of some of the most common self-labeling tags include SNAP-tag, Halo-tag, and CLIP-tag.

Unlike FPs, self-labeling enzymes are not naturally fluorescent and therefore must be linked to a fluorescent dye (Stagge et al., 2013). This is achieved through the use of a substrate linker. Firstly, the POI is genetically fused to the self-labeling tag. As each of the enzymes binds to a fluorescently conjugated substrate, this substrate can then covalently react, successfully tagging the POI for fluorescence microscopy (Keppler et al., 2003).

The Halo-tag originates from the bacterial enzyme haloalkane dehalogenase and is modified to ensure it can covalently bond to synthetic ligands. The enzyme can be conjugated with the POI through the substrate linker chloroalkane (Los et al., 2008). The Halo-tag is able to fuse with the chloroalkane linker through the removal of the chlorine atom through a nucleophilic displacement mechanism (England et al., 2015). Similarly, the SNAP-tag and CLIP-tag are both modified forms of the human enzyme O^6^-alkylguanine-DNA alkyltransferase (hAGT) with the former covalently interacting with O^6^-benzylguanine derivatives and the latter so with the O^2^-benzylcytosine derivatives (Stagge et al., 2013). Both of these compounds act as substrates to their respective mutant hAGT enzymes (Hoehnel and Lutolf, 2015). These tags similarly form an irreversible covalent bond with their substrate that links the fluorophore.

Factors that make self-labeling tags advantageous over FPs are prolonged observation time through limiting photobleaching and the ability to emit more photons in blinking periods and therefore providing improved brightness of the fluorophore (Li and Vaughan, 2018; Banaz et al., 2019). Furthermore, the tags are also more open to modification without the loss of fluorescence when compared to FPs. Therefore, the features of these tags are aimed to improve upon the limitations presented by FPs (Yan and Bruchez, 2015).

However, despite improvements in flexibility and brightness, self-labeling tags continue to present disadvantages in SMLM. Like FPs, self-labeling tags require the enzymes to be genetically fused to the POI. This can similarly cause perturbations to protein-protein interactions due to its larger size (Fernández-Suárez and Ting, 2008). It also lacks the capability of site-specific protein tagging which can be beneficial when wanting to image specific regions of the POI (Lee et al., 2019). Therefore, for finding an appropriate fluorescent label for SMLM, it could be beneficial to find alternatives in using genetic in-fusion proteins.

## Unnatural Amino Acids as a Viable Alternative

### Unnatural Amino Acids

The limitations presented by the use of FPs and other fluorophore labeling strategies have influenced the search for more ideal candidates for fluorescent tagging. Amongst the labeling strategies in practice for SMLM imaging, an upcoming technique is the use of fluorescently labeled UAAs, also referred to as the expanded genetic code (Saal et al., 2018). The genetic code is made up of 20 standard amino acids which make up the natural building blocks of life, however, hundreds of amino acids outside of this have been discovered in nature (Brown et al., 2018). Unnatural amino acids (UAAs) thus are structures that are not found in natural proteins but can be found in other parts of nature or can be chemically synthesized (Wiltschi, 2016).

**Figure 2:**
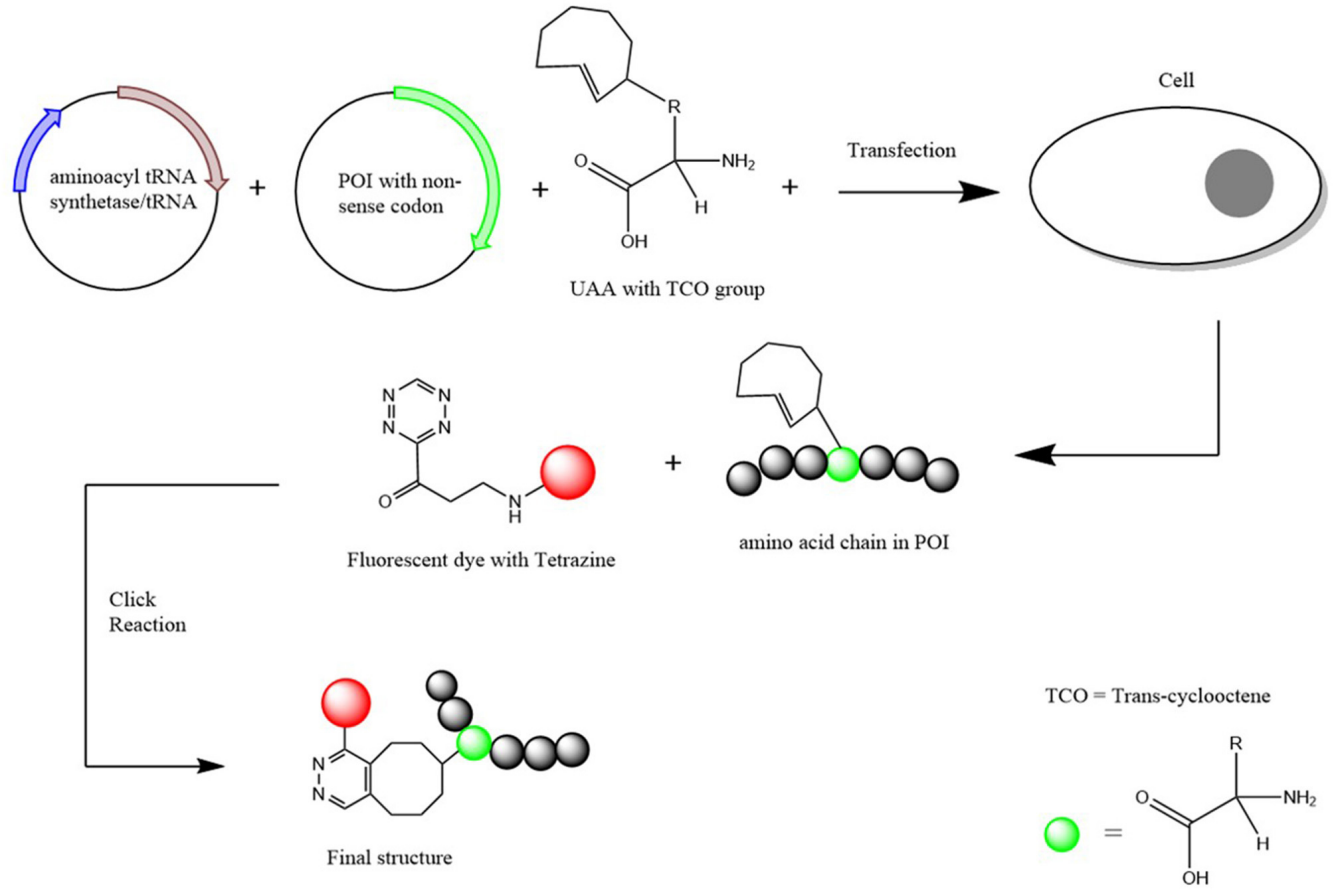
Schematic Diagram of how an UAA is incorporated into a protein of interest. Two plasmids (one containing the aminoacyl-tRNA synthetase and the other the POI with the non-sense codon inserted) and the UAA with a TCO group attached are transfected into the chosen cells. Once incorporated, a fluorescent dye with a tetrazine group is added for the click chemical reaction to result in a structure that attaches the POI with the fluorescent dye together. Scheme based on Nikić et al. (2016).

The expansion of the genetic code through the use of UAAs have become a common practice in many biomolecular applications as it continues to show they aid in the better understanding in the functioning of proteins and other biomolecules (Stevenazzi et al., 2014). Unnatural amino acids (UAAs) have also been found to be advantageous in many biological/chemical applications such as drug discovery and protein labeling (Narancic et al., 2019). Along with the standard amino acids, two additional amino acids have been incorporated into biological systems which are selenocysteine (Sec) and pyrrolysine (Pyl) and these are at times referred to as the 21^st^ and 22^nd^ amino acids (Ambrogelly et al., 2007) (Figure 2).

### UAA Incorporation

The site-specific incorporation began through the discovery that the two extra amino acids Sec and Pyl that correspond to the opal (TGA) and amber (TAG) codons, respectively (Böck et al., 1991; Krzycki, 2005). This is done through the aid of a corresponding amino acid insertion sequence that is able to suppress the stop codon and subsequently add the amino acid (Xue et al., 2015). Through this discovery, it could be shown that the natural translational mechanism can incorporate UAAs into a POI. This therefore led to the discovery of the insertion of other UAAs through the use of orthogonal aminoacyl-tRNA synthetase/tRNA pairs (Schmied et al., 2014). Various forms of the orthogonal pairs have been created depending on the choice of amino acid that would be incorporated (Cervettini et al., 2020). Through the use of a non-sense codon and the orthogonal pair selective to the fluorescent UAA, hundreds of UAAs can now be incorporated into a particular protein/polypeptide chain (Jakob et al., 2019). This method of UAA incorporation is known to be highly selective and kinetically fast so therefore has potential to be the most preferred method of UAA incorporation over other biochemical strategies (Pantoja et al., 2009).

Once incorporated into the POI, the UAA is then fluorescently labeled for SMLM imaging. Labeling is achieved by the addition of chemical handles to the amino acid through a bio-orthogonal “click” reaction (Courtney and Deiters, 2018). This can be done through an azide-alkyne cycloaddition with Copper [Cu (I)] as the needed catalyst. The Cu(I) catalyzed reaction provides the benefits of kinetically fast reaction times, great regioselectivity and high yields, however, poses a threat to cells due to the toxic nature of the copper catalyst (Baskin et al., 2007). To overcome the toxic nature of the copper catalyst, an alternative copper free reaction is used for the click reaction (Gutmann et al., 2016). However, the alternative reaction does come with its own issues regarding efficacy causing the yield to potentially be a lot lower than its catalytic version. Therefore, while the alternative copper free reaction is a preferred method to overcome toxicity, reaction times may need to be extended to ensure more complete conjugation of the fluorescent label to the UAA (Li and Zhang, 2016).

### UAAs as Fluorescent Tags

One primary benefit of the usage of UAAs include the site-specific placement of the fluorescent tag unlike the usage of other labeling techniques. This can allow for the monitoring of protein interactions at the desired location producing more localized data for single molecules as FPs and other similar fluorophores are required to be genetically fused to the C or N terminus (Summerer et al., 2006). Unnatural amino acids (UAAs) are often also easier to tag onto the gene/POI due to its significantly smaller size when compared to larger FPs (Neumann-Staubitz and Neumann, 2016).

Choosing the appropriate amino acid to incorporate in SMLM would be based on a synergy of fluorescence levels, ease of insertion and its ability in reversibly photoswitching. A popular strategy is incorporating the desired amino acid with a plasmid containing the GFP gene. These along with the plasmid for the orthogonal tRNA synthetase pair can provide the necessary fluorescence for imaging (Schmied et al., 2014). Other examples of this technique used with many various types of UAAs have been used and have shown high fluorescence and efficiency (Hino et al., 2005; Nikić et al., 2016; Hilaire et al., 2017). This can be compared with studies using different fusion proteins to highlight the subtle improved quality of fluorescently tagged UAA and the disadvantages posed with the use of the bulky fluorophores (Cinelli et al., 2000; Snapp et al., 2003; Wang et al., 2014). As the field continues to advance, different types of UAAs are continuing to be successfully developed in an effort to achieve the ideal imaging of biomolecule constructs.

The first reported single-molecule imaging of a fluorescently labeled UAA was done by Pantoja et al. (2009). In this report total internal reflection fluorescence microscopy was used to image individual molecules of a fluorescent lysine conjugate UAA incorporated into muscle nicotinic acetylcholine receptor in *Xenopus* oocytes. This report provided the first proof of concept which has been applied to other microscopy methods including super resolution techniques. Uttamapinant et al. (2015) utilized the incorporation of fluorescently labeled UAAs in observing live cells through the use of STORM imaging. The study managed to visualize structures beyond the diffraction limit and have successfully generated precise images of the biomolecules. Similar super-resolution techniques that utilize UAA incorporation (Sakin et al., 2017; Schvartz et al., 2017; Neubert et al., 2018) also demonstrate the benefits of their use. However, as the area remains relatively new and up and coming, the number of successful studies using SMLM to visualize nanoscopic biomolecules remains limited.

## Conclusion

Single Molecule Localization Microscopy has revolutionized the field of biomolecule imaging due to its ability to investigate cellular and protein structures that go beyond the diffraction limit that regular light microscopy lacks to do. However, the most successful experiments involve the use of the best type of fluorophores labeled to the molecules. While many common fluorescent tags have been used in the past such as FPs and self-labeling tags, these come with their own limitations. In an attempt to combat these, UAA can be incorporated into the gene/POI to provide better fluorescence and therefore more precise imaging in SMLM. The use of UAAs as tags in SMLM have shown to have several benefits such as site-specific incorporation and the lower likelihood of the hinderance of intrinsic protein–protein interactions due to its much smaller size. Despite these benefits, due to the limited number of studies available, the practical aspects of UAAs as probes needs further study. For example, the efficacy of certain reactions used such as the copper-based cycloaddition or in the transfection process involving the UAA and target constructs may vary between cells of interest. Despite these technical challenges, the added benefits of targeted, site specific labeling with UAA promises to unlock the full potential of super-resolution microscopy.

## Author Contributions

PL has first authorship while authors SA, KG, and JG all share senior authorship. All authors contributed to the article and approved the submitted version.

## Conflict of Interest

The authors declare that the research was conducted in the absence of any commercial or financial relationships that could be construed as a potential conflict of interest.
